# Disposable diaper overuse is associated with primary enuresis in children

**DOI:** 10.1038/s41598-020-70195-8

**Published:** 2020-09-01

**Authors:** Xing Li, Jian Guo Wen, Tong Shen, Xiao Qing Yang, Song Xu Peng, Xi Zheng Wang, Hui Xie, Xing Dong Wu, Yu Kai Du

**Affiliations:** 1 Department of Maternal and Child Health, School of Public Health, Tongji Medical College, Huazhong University of Science & Technology, 13 Hangkong Rd, Wuhan, 430030 Hubei China; 2 Department of Urology, The First Affiliated Hospital of Zhengzhou University, Zhengzhou, 450002 China; 3 Department of Pediatric, Women and Children’s Hospital, School of Medicine, Xiamen University, Xiamen, 361000 China

**Keywords:** Risk factors, Paediatric urology

## Abstract

This research investigated the association between prolonged disposable diaper (DD) wearing in infancy and primary enuresis (PNE). As a case–control study, we collected data from 376 children with enuresis and 379 healthy children who were sex- and age-matched at three tertiary care institutions in mainland China from August 2017 to July 2018. The results of adjusted logistic regression showed the odds ratios (95% confidence intervals) for PNE across the categories of age of daytime DD use cessation were as follows: ≥ 25 months: 1.00, 18–24 months: 0.25 (0.17–0.37), and ≤ 17 months: 0.11 (0.06–0.20), independent of age, mother education, residence, toilet training approach, breastfeeding duration, UTI, constipation, anaphylactic disease and family history. After a similar multivariable adjustment, increased age of daytime DD use (per-month) had a positive correlation with PNE, OR = 1.17, 95% CI 1.13–1.20 and non-linear relationship was detected, whose point was 21 months (the effect sizes and the 95%CI on the left and right sides of inflection point were 1.04 (0.99–1.10), *P* = 0.131 and 1.25 (1.18–1.31), *P* < 0.001). Subgroup analysis found that the effect of duration of disposable diaper exposure for each additional month, those children had accepted assisted infant toilet training/elimination communication (AITT/EC) practice had a lower risk of PNE (OR = 1.08, 95% CI 1.04–1.12), compared with those without AITT/EC practice (OR = 1.20, 95% CI 1.14–1.27), *P* for interaction < 0.001. In conclusion, the children diagnosed with primary enuresis after age 5 stopped using disposable diapers at daytime later than the control group. Association between duration of DD exposure and the risk of childhood enuresis is modified by AITT/EC practice. Timely cessation use of disposable diaper and practice AITT/EC may shorten the time to nocturnal continence, and the prospective cohort studies are needed to verify the discoveries.

## Introduction

Primary enuresis is characterized by intermittent incontinence in children 5 years or older during sleep without any history of being dry for more than 6 months^1^. It has a negative psychological and social impact on the child and his or her family and brings a larger disease burden as well^2^. Primary enuresis occurs in neurologically normal children and is considered a functional voiding disorder due to delayed brain-bladder function development. The disorder is multifactorial, and genetic predisposing factors, delays in attaining bladder control, the circadian rhythm of vasopressin and psycho-social factors may all contribute to its development^3^. However, the underlying pathophysiologic mechanisms are still not fully understood.

The overall prevalence of childhood enuresis varies from 2.3 to 25%^2–6^. Liu reported a prevalence of 4.3% in mainland China in 1997, which was markedly lower than that reported in Western countries during the same period^5^. Our team conducted an epidemiological study on Chinese children and adolescents in 2006 and 2017 respectively, and found that the overall prevalence of primary enuresis increased significantly in 2017 compared to 2006 (7.30% versus 4.07%, *P* < 0.001), with a prevalence of approximately 11.83% in 5-year-old children in 2006 and 15.13% in the same age group in 2017^6,7^. One noticeable change over the decades has been the introduction of disposable diapers (DDs) in China, leading to a decline in toilet training (TT) and overreliance on disposable diapers by parents before the children reach 2.5–4 years of age.

Assisted infant toilet training (AITT) or elimination communication (EC) refers to a parenting practice that involves learning infants’ elimination signal and schedules and physically assisting infants during voiding and bowel movements, starting in early infancy^8^. In fact, this approach was widely used in China a decade ago but is now being gradually replaced by disposable diapers, and the caregiver’s attitude towards AITT was affected by internet and We-media platform, argument continued, and public perception is confused, leading to an inconsistency between attitude and practice.

The age at which toddlers become diaper-free has increased from 1.5–2 years of age in the 1950s to 3–3.5 years of age currently worldwide^9–13^. The age initiation of bladder training remains controversial in the pediatric field. In previous decades, some investigators believed that the development of bladder control was largely a maturational process and could not be accelerated by early and high intensity toilet training and that attempts to accelerate toilet training were a waste of time^14,15^. On the other hand, many investigators have argued that the current trend toward delayed toilet training may not continue and that it is possible that delaying the onset of toilet training prolongs the exposure time to potential stressors; there might also be a relation between late bladder training and dysfunctional bladder^16–20^.

In the current multicenter retrospective study, we sought to investigate AITT/EC practice and the age of daytime disposable diaper use cessation in children with primary nocturnal enuresis to examine whether associations existed between disposable diaper dependence (DDD) and childhood enuresis**.**

## Results

### Demographic and baseline characteristics of the study population

In this multicenter study, 430 children who received care at our institutions for enuresis were enrolled. Among them, 27 cases were excluded because of neurogenic bladder, and 16 were excluded due to combined developmental retardation (12) and organic kidney diseases (4). A total of 387 questionnaires were collected, and 97% of them were complete. Finally, 376 children with primary enuresis were included. Their mean age was 6.79 ± 1.92 years (range 5 to 15 years), and 229 (60.4%) of them were boys. Furthermore, 379 healthy age- and sex-matched children were included in the control group.

The median age of daytime disposable diaper use cessation was 26 (range 24 to 32) months in children with primary enuresis and was significantly delayed compared with that of the control group (median 20 months; range 18 to 25 months, *P* < 0.001). The data for age of cessation of disposable diaper use were classified into three categories: ≤ 17 months, 18–24 months and ≥ 25 months, and the Mann–Whitney test showed statistical differences between the PNE and control groups, *P* < 0.001. In the control group, 297 (78.4%) children had undergone daytime AITT or EC (elimination communication before 18 months), and only 157 (41.8%) in the PNE group had undergone daytime AITT/EC. The demographic baseline characteristics of disposable diaper use and AITT/EC practice of the study population are shown in Table 1.

**Table 1:** Baseline and demographic characteristics of the study population.

Variables	PNE	Controls	*P*
**N**	376	379	
**Baselines**			
Mean age (SD), years	6.79 ± 1.92	6.88 ± 2.05	0.524
Boys, N (%)	229 (60.4)	229 (60.9)	0.941
Urban resident, N (%)	257 (68.4)	283 (74.7)	0.064
Mother's education, years, N (%)			< 0.001
≤ 9	183 (48.7)	128 (33.8)	
> 9	193 (51.3)	251 (66.2)	
**Feeding, N (%)**			0.287
Exclusive breastfeeding	178 (47.3)	198 (52.3)	
Bottle feeding	44 (11.7)	34 (9.0)	
Mix	154 (41.0)	147 (38.8)	
Breastfeeding duration < 6 M	140 (37.2)	84 (22.2)	< 0.001
**Others related factors**			
Anaphylactic disease, N%	44 (11.7)	37 (9.8)	0.412
Family history, N%	82 (21.8)	9 (2.5)	< 0.001
**Bladder and bowel symptoms**			
LUTs	118 (31.4)	46 (12.1)	< 0.001
Constipation	74 (19.8)	50 (13.2)	0.016
**TT and duration of DD exposure**			
AITT/EC attitude (yes, N%)	190 (50.5)	241 (63.6)	< 0.001
EC (AITT) practice	157 (41.8)	297 (78.4)	< 0.001
**TT approach**			0.281
Waiting	38 (10.1)	26 (6.9)	
Strict	14 (3.7)	9 (2.4)	
Interaction	216 (57.4)	222 (58.6)	
Regular	108 (28.7)	122 (32.2)	
**Age of DD use cessation, M (IQR), months**	26 (24,32)	20 (18,25)	< 0.001
≤ 17, N%	23 (6.1)	92 (24.3)	
18–24, N%	119 (31.6)	190 (50.1)	
≥ 25, N%	234 (62.2)	97 (25.6)	
**Daytime Bladder Control, M(IQR), months**	30 (25,36)	24 (20,29)	< 0.001
≤ 24, N%	93 (24.7)	224 (59.1)	
> 24, N%	283 (75.3)	147 (38.8)	

*LUTS* lower urinary tract symptoms, *TT* toilet training, *AITT* assisted infant toilet training, *DD* disposable diaper, *M(IQR)* median (interquartile range).

### The results of multivariate and subgroup analyses association with primary enuresis

Univariate odds ratios are presented in Table 2. The non-adjusted and adjusted association between duration of DD exposure and PNE is presented in Table 3. After adjusting potential confounders, increased DD use age (per-month) had a positive correlation with PNE, odds ratio = 1.17, 95% confidence interval (CI) 1.13–1.20, *P* for trend < 0.001. The multivariable adjusted odds ratios (95% CI) for enuresis across the categories of age of daytime DD use cessation were as follows: in crude model, ≥ 25 months: 1.00, 18–24 months: 0.26 (0.19–0.36), ≤ 17 months: 0.10 (0.06–0.17), in model 2, ≥ 25 months: 1.00, 18–24 months: 0.37 (0.25–0.54), ≤ 17 months: 0.19 (0.11–0.36), independent of age (years), mother’s education (years), residence, breastfeeding duration (months), constipation, anaphylactic disease, family history, AITT/EC attitudes, AITT/EC practice and TT approach; in model 3, Model 2 removed the covariate of AITT/EC practice, showed: ≥ 25 months: 1.00, 18–24 months: 0.25 (0.17–0.37), ≤ 17 months: 0.11 (0.06–0.20), respectively. The results indicated after progressive adjustment for various relevant covariates, the age of DD use cessation at daytime remained significantly associated with PNE. Comparing with DD use cessation ≥ 25 months, the risk of developing PNE at categories of age DD use cessation between 18–24 months and ≤ 17 months were reduced by 63% and 81%, respectively (including adjustment of AITT/EC practice); while without adjustment of AITT/EC practice, compared with DD use cessation ≥ 25 months, DD use cessation between 18–24 months and ≤ 17 months, reduced PNE risk by 75% and 89%, respectively (Table 3).

**Table 2:** The results of univariate logistic regression analysis for factors associated with enuresis.

Variabilities	Statistics	Effect size (ORs)	*P* value
**Age of daytime DD use cessation (months)**	23.40 ± 7.77	1.15 (1.12–1.18)	< 0.001
≥ 25	331 (43.84%)	1	
18–24	309 (40.93%)	0.26 (0.19–0.36)	< 0.001
≤ 17	115 (15.23%)	0.10 (0.06–0.17)	< 0.001
**Sex**			
Boys	458 (60.66%)	1	
Girls	297 (39.34%)	0.98 (0.73–1.31)	0.892
**Age (years)**	6.83 ± 1.98	0.98 (0.91–1.05)	0.524
**Mother's education (years)**			
≤ 9	311 (41.19%)	1	
> 9	444 (58.81%)	0.54 (0.40–0.72)	< 0.001
**Residence**			
Urban	540 (71.52%)	1	
Suburb	215 (28.48%)	1.36 (0.99–1.88)	0.055
**AITT attitude**			
Support	431 (57.09%)	1	
Against	324 (42.91%)	1.71 (1.28–2.29)	< 0.001
**AITT practice**			
No	301 (39.87%)	1	
Yes	454 (60.13%)	0.20 (0.14–0.27)	< 0.001
**TT approach**			
Waiting	64 (8.48%)	1	
Strict	23 (3.05%)	1.06 (0.40–2.827)	0.900
Interaction	444 (58.81%)	0.68 (0.40–1.51)	0.152
Regular	224 (29.67%)	0.58 (0.33–1.02)	0.060
**UTI**			
No	719 (95.23%)	1	
Yes	36 (4.77%)	2.74 (1.30–5.77)	0.008
**Constipation**			
No	631 (83.58%)	1	
Yes	124 (16.42%)	1.61 (1.09–2.38)	0.017
**Anaphylactic disease**			
No	674 (89.27%)	1	
Yes	81 (10.73%)	1.23 (0.77–1.95)	0.390
**Family history**			
No	664 (87.95%)	1	
Yes	91 (12.05%)	11.47 (5.67–23.21)	< 0.001
**Breastfeeding duration**	8.62 ± 4.01	0.97 (0.94–1.01)	0.135
> 6	531 (70.33%)	1	
≤ 6	224 (29.67%)	2.08 (1.51–2.87)	< 0.001

*DD* disposable diaper, *AITT* assisted infant toilet training, *TT* toilet training, *UTI* urinary tract infection.

**Table 3:** Adjusted odds ratios (with 95% confidence intervals) from logistic regression models for the association of age of daytime disposable diaper use cessation with enuresis.

Exposure	Model 1	Model 2	Model 3
	Odds ratio (95% CI)	Odds ratio (95% CI)	Odds ratio (95% CI)
DD use cessation age (months)	1.15 (1.12–1.19) < 0.001	1.13 (1.09–1.17) < 0.001	1.17 (1.13–1.20) < 0.001
**DD use cessation age groups (months)**			
≥ 25	1	1	1
18–24	0.26 (0.19–0.36) < 0.001	0.37 (0.25–0.54) < 0.001	0.25 (0.17–0.37) < 0.001
≤ 17	0.10 (0.06–0.17) < 0.001	0.19 (0.11–0.36) < 0.001	0.11 (0.06–0.20) < 0.001
P for trend	0.30 (0.24–0.38) < 0.001	0.42 (0.31–0.56) < 0.001	0.30 (0.23–0.40) < 0.001

Model 1: Crude model, adjust for: None. Model 2: model 1 adjusted for: age (years); education (years); residence; breastfeeding duration (months); family history; anaphylactic disease; constipation; UTI; AITT attitude; AITT practice and TT approach; Model 3: adjusted for: age (years); education (years); residence; breastfeeding duration (months); family history; anaphylactic disease; constipation; UTI, AITT attitude and TT approach.

*DD* disposable diaper, *UTI* urinary tract infection, *AITT* assisted infant toilet training, *TT* toilet training.

Additionally, we performed a subgroup analyses and found that the effect of duration of disposable diaper exposure on PNE was significantly influenced by AITT/EC practice. Compared with participants without AITT/EC practice (OR = 1.20, 95% CI 1.14–1.27), odds ratio for participants with AITT/EC practice was 1.08, 95% CI 1.04–1.12, *P* < 0.001, *P* for interaction < 0.001 (Table 4). AITT/EC practice is a protective factor for PNE.

**Table 4:** Effect size of Daytime DD use cessation age on Enuresis and exploratory subgroups.

Characteristic	No of participants	Effect size (95% CI)	*P* for interaction
**Residence**			0.070
Urban	540	1.19 (1.15, 1.23)	
Suburb	215	1.11 (1.07, 1.16)	
**Mother's education (years)**			0.222
≤ 9	311	1.14 (1.09, 1.18)	
> 9	444	1.17 (1.13, 1.22)	
**Sex**			0.200
Male	458	1.14 (1.10, 1.18)	
Female	297	1.18 (1.13, 1.24)	
**Age (years)**			0.730
≥ 8	222	1.17 (1.11, 1.23)	
6–7	323	1.13 (1.09, 1.18)	
5	210	1.19 (1.12, 1.26)	
**AITT attitude**			0.008
Support	431	1.13 (1.10, 1.17)	
Against	324	1.19 (1.13, 1.24)	
**AITT practice**			< 0.001
No	301	1.20 (1.14, 1.27)	
Yes	454	1.08 (1.04, 1.12)	
**UTI**			0.060
No	719	1.15 (1.12, 1.18)	
Yes	36	1.63 (1.08, 2.46)	
**Constipation**			0.851
No	631	1.15 (1.12, 1.19)	
Yes	124	1.15 (1.08, 1.24)	
**Anaphylactic disease**			0.324
No	674	1.17 (1.13, 1.20)	
Yes	81	1.09 (1.02, 1.17)	
**Family history**			0.216
No	664	1.16 (1.12, 1.19)	
Yes	91	1.14 (1.01, 1.29)	
**TT approach**			0.121
Waiting	64	1.25 (1.13, 1.39)	
Strict	23	1.05 (0.95, 1.17)	
Interaction	444	1.18 (1.14, 1.23)	
Regular	223	1.12 (1.07, 1.18)	
**Breastfeeding duration (months)**			0.080
> 6	531	1.17 (1.13, 1.21)	
≤ 6	224	1.11 (1.07, 1.17)	

*DD* disposable diaper, *AITT* assisted infant toilet training, *TT* toilet training, *UTI* urinary tract infection.

### The results of threshold effect analysis

**Figure 1:**
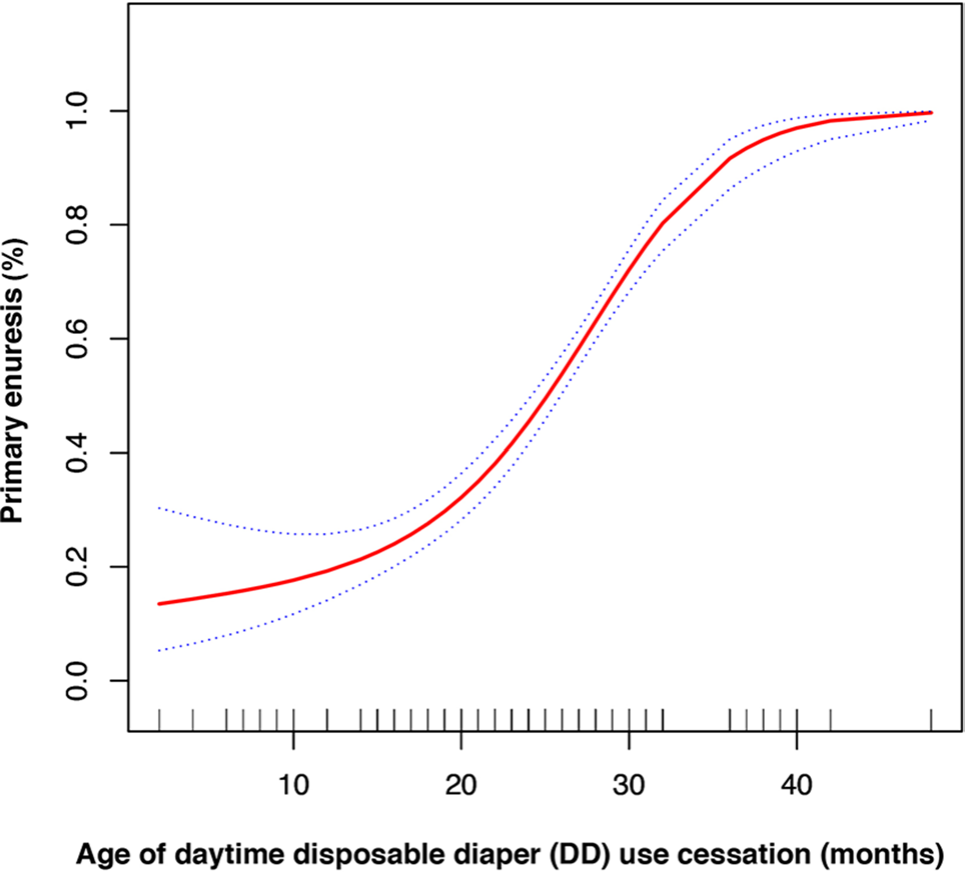
Association between the age of disposable diaper use cessation and primary enuresis (%). A nonlinear relationship between them was detected after adjusting for age (years), mother’s education (years), residence, UTI, constipation, anaphylactic disease and family history.

In the present study (Fig. 1), we found that the association between age of DD use cessation and PNE was non-linear (after adjusting for age, mother's education, residence, UTI, constipation, anaphylactic disease and family history). By two-piecewise regression model, we calculated the inflection point was 21 (months). On the right of inflection point, the effect size (OR), 95% CI and *P* value were 1.25, 1.18–1.31 and 0.001, respectively. However, we observed no association between age of daytime DD use cessation and PNE on the left of inflection point (1.04, 0.99–0.10, 0.131) (Table 5 and Fig. 1).

**Table 5:** Threshold effect analysis of duration of DD use on PNE using piece-wise regression.

Inflection point of age of daytime DD use cessation (months)	Effect size (OR)	95% CI	*P* value
≤ 21	1.04	0.99–1.10	0.131
> 21	1.25	1.18–1.31	< 0.001

Effect: Primary enuresis; Cause: Age of DD use cessation. Adjusted: age(years); mother’s education(years); residence; UTI; constipation; anaphylactic disease; family history.

*DD* disposable diaper, *UTI* urinary tract infection.

## Discussion

The present study provided compelling evidence on the link between prolonged daytime disposable diaper usage and childhood enuresis, the association is modified by AITT/EC practice and children benefit from infancy AITT/EC practice for achieving nighttime bladder control. However, we also found the different correlations of the age of daytime DD use cessation on PNE on the left and right sides of inflection point (age of DD use cessation = 21 months). The age of DD use cessation was positively associated with PNE on the right side of inflection point, but the association on the left of inflection was not statistically significant.

Previous researches had indicated that childhood nocturnal enuresis is an interaction of genetic predisposition and the nurturing environment, supported the coexistence of enuresis and constipation, therefore, we adjusted the covariates of family history and constipation in the final model for an alternative mechanism.

The American Academy of Pediatrics (AAP) has published the “Guide to Toilet Training”^21,22^ and recommended that toilet training should be initiated when children show physical and emotional readiness to train, which usually emerges at age 18–24 months or later. However, the guidelines also pointed out that initiating training before 18 months is unlikely to do any damage as long as your expectations for your child’s performance are realistic and no punishment or abuse is involved.

Recent research has indicated that childhood enuresis mostly occurs because the brain does not respond to the bladder’s signal that it is full^23,24^. We speculate that bedwetting may be due to years of practice sleeping with disposable diapers, which conditions the brain to ignore the bladder. By eliminating the sensation of wetness, disposable diapers may impact bladder-brain links^25,26^. Primary nocturnal enuresis occurs in either the rapid eye movement or nonrapid eye movement stage of sleep, and children are known to have spontaneous and induced detrusor contraction^24,27^. Although the mechanisms are still not entirely clear, delayed development of urination control centers may be one of the culprits because in many children, enuresis disappears with increasing age. Its incidence is related to the establishment of a conditioned reflex and heredity. In general, when a baby's bladder fills up, it stimulates the cerebral cortex to start voiding, and the external sphincter of the urethra can be autonomously used to control urination in children aged 2 to 3 years. If the autonomic urination reflex is interrupted, enuresis ensues. Extended use of disposable diapers could deny the neurological pathways the chance to form and create an autopilot^28^. Meanwhile, by eliminating the sensation of wetness, disposable diapers cause insensitivities and delay the establishment of a conditioned reflex.

Previous studies have found that the connections between the brain and bladder are already established in newborns, that there is cortical arousal in response to a full bladder, even in newborn infants^25,26^. AITT/EC, which has been widely practiced by Chinese caregivers, uses the infant’s natural timing and cues to recognize the need to defecate or urinate. By identifying these cues, caregivers can coordinate elimination in the toilet rather than in a diaper. Response signals generated by the full bladder stimulate a child’s perception of bladder filling ability and may accelerate the sphincter to adjust learning of active control^29,30^.

Liu et al.^5^ showed that Chinese children attained nocturnal urinary control earlier than Western children; the prevalence of nocturnal enuresis was low but fairly stable in children between 6 and 16 years of age in 1997. Bakker et al*.*^17^ reported that youngsters who had bladder problems at age eleven started toilet training after two years of age. Akis^31^ found an unadjusted odds ratio for gained toilet training before 18–24 months would get enuresis 3.04 times less than one who has gained toilet training later. Yang et al*.*^19^ evaluated urinary continence status and bladder function in healthy kindergartener, revealed early initiation of toilet training for urine was associated with early urinary continence and does not appear to be associated with bladder dysfunction and Joinson et al.^18^ observed the evidence that initiating toilet training after 24 months is associated with problems attaining and maintaining bladder control. Our study confirms the previous results and indicates the independent effect by control the relevant covariates (e.g., constipation and allergic disease).

Inconsistent with our findings, some investigators argued that lower urinary tract dysfunction is closely associated with incorrect or early toilet training, which is caused by parental expectations that do not consider the child’s neurological development stage^14,15^. However, although the mainstream toilet training time has been delayed worldwide in recent decades, the prevalence of LUT dysfunction and bowel dysfunction is reported to be on the rise.

Most notably, to our knowledge, this is the first study of the relationship between disposable diaper overuse and childhood nocturnal enuresis. We are aware of some limitations of this study. First, although the study is multicenter, our subjects were ethnically and geographically limited due to the study design as a hospital-based case–control study that is observational in nature and focuses on a functional disorder. Second, as a retrospective study, biases could not be avoided completely during the collection of data.

In conclusion, our study has demonstrated that the children diagnosed with primary enuresis after age 5 stopped using disposable diapers at daytime later than the control group. Association between duration of DD exposure and the risk of childhood enuresis is modified by AITT/EC practice. Timely cessation use of disposable diaper and practice AITT/EC may shorten the time to nocturnal continence, and this may or may not be a causal association, the prospective cohort studies are needed to verify our discoveries.

## Methods

This retrospective case–control study included children with primary enuresis who received medical care at our Enuresis Clinics at the Women’s and Children’s Hospital, Xiamen, the First Affiliated Hospital of Zhengzhou University, Zhengzhou, and Shenzhen Children’s Hospital, Shenzhen, China, between August 2017 and July 2018. In addition, sex- and age-matched healthy children who underwent wellness examinations at the hospital in the same period were also enrolled (Supplementary Fig. 1, study flowchart).

Primary enuresis was diagnosed according to the International Children’s Continence Society (ICCS) criteria^1^. Children 5 years or older who had intermittent incontinence during sleep and no history of being dry for more than 6 months were enrolled.

Children with a neurogenic bladder, urologic and orthopedic malformations or congenital or neurological diseases and diseases associated with childhood development were excluded.

The study protocol was approved by the human research ethics committees of Women and Children’s Hospital, School of Medicine, Xiamen University. Informed consent from the parents or legal guardians of participating children was obtained. All methods were performed in accordance with the relevant guidelines and regulations.

### Primary enuresis questionnaire

The authors developed a structured questionnaire, which was further revised by pediatric urologists, child behavior development specialists and kindergarten educators (Supplementary Material 2). The questionnaire included information on (1) demographic characteristics, such as age, sex, residence area and mother’s educational level; (2) caregiver attitudes, nurturing behaviors and practices of toilet training and disposable diaper usage; ((3) severity of primary nocturnal enuresis and concurrent lower urinary tract symptoms (LUTSs) and elimination disorders; (4) family history; and (5) other factors associated with nocturnal enuresis such as breastfeeding and an allergic disease history. The questionnaire was answered by the mothers during the hospital visit. The collected questionnaires were further checked by data managers to ensure the integrity of the information. The parents were contacted via telephone by investigators if the returned questionnaire contained ambiguous answers. A questionnaire was considered completed if the main points were completely answered. We also collected the medical records, including laboratory examinations of the children at the same time to confirm the diagnosis.

Elimination communication is referred to as assisted infant toilet training (AITT or EC)^8,32^, defined as the process of a caregiver assisting and enabling a child to meet his or her basic cleanliness and health needs for toileting from early infancy (before 18 months) via verbal and nonverbal communication, or the child started using the potty regardless of the frequency of potty use. Disposable diaper use cessation was the period of cessation use of diapers at the discretion of the caregiver and the start of intensive toilet training. Bladder control was achieved when the child was aware of the need to void, expressed their need via verbal and nonverbal communication, stayed dry and had no retention of urine.

### Statistical analysis

All continuous variables were expressed as the mean ± standard deviation or the median and interquartile range and compared using an unpaired Student’s t test or the nonparametric Mann–Whitney test. Categorical variables were presented as counts and percentages and compared using chi-square statistics.

According to the AAP “Guide to Toilet Training”, toilet training can be started at the age of 18–24 months when children show physical and emotional readiness; we classified the data for age of daytime disposable diaper use cessation (start intensive toilet training) into three categories for further analysis: ≤ 17 months, 18–24 months and ≥ 25 months.

We used PNE as dependent variables and the categories of age disposable diaper use cessation as independent variables, odds ratios (ORs) and a 95% confidence interval (95% CI) for PNE were calculated. Whether the covariates were adjusted determined by the following principle: when added to this model, changed the matched odds ratio by at least 10%, or clinically relevant, or were significantly associated with PNE. The effects of age of DD use cessation categories on childhood enuresis were evaluated with the use of binary logistic regression models with adjusted for age, mother education, residence, UTI, constipation, anaphylactic disease, family history. Subgroup analyses were performed using stratified logistic regression models. The modification and interaction of subgroup were inspected by the likelihood ration test. Besides, since the age of daytime DD use cessation was a continuous variable, the analyses of nonlinear relationship are necessary. We used generalized additive model (GAM) to identify the non-linear relationship. If the non-linear correlation was observed, a two-piecewise regression model was performed to calculate the threshold effect of the age DD use cessation on PNE in terms of the smooth curve fitting. When the ratio between PNE and age DD use cessation appears obvious in smoothed curve, recursive method calculates automatically the inflection point, where the maximum model likelihood will be used. A *P* value < 0.05 was considered statistically significant in all analyses.

Data were analyzed with the use of the statistical packages R (The R Foundation; https://www.r-project.org; version 3.4.3 2018-02-18) and EmpowerStats (https://www.empowerstats.com; X&Y Soluntions Inc.).

## Supplementary information

Supplementary Information.

## Data Availability

The datasets generated during and/or analyzed during the current study are available from the corresponding author on reasonable request.

## References

[1] Austin PF (2016). The standardization of terminology of lower urinary tract function in children and adolescents: Update report from the standardization committee of the International Children's Continence Society. Neurourol. Urodyn..

[2] Tai TT, Tai BT, Chang YJ, Huang KH (2017). Parental perception and factors associated with treatment strategies for primary nocturnal enuresis. J. Pediatr. Urol..

[3] Arda E, Cakiroglu B, Thomas DT (2016). Primary nocturnal enuresis: A review. Nephro-Urol. Month..

[4] Yeung CK, Sreedhar B, Sihoe JD, Sit FK, Lau J (2006). Differences in characteristics of nocturnal enuresis between children and adolescents: A critical appraisal from a large epidemiological study. BJU Int..

[5] Liu X, Sun Z, Uchiyama M, Li Y, Okawa M (2000). Attaining nocturnal urinary control, nocturnal enuresis, and behavioral problems in Chinese children aged 6 through 16 years. J. Am. Acad. Child Adolesc. Psychiatry.

[6] Wen JG, Wang QW, Chen Y, Wen JJ, Liu K (2006). An epidemiological study of primary nocturnal enuresis in Chinese children and adolescents. Eur. Urol..

[7] Wang, X. Z., Wen, J. G. & Wen, Y. B. The impact of using diapers on children's enuresis in Zhengzhou China. In Florence: ICS 2017 Paediatrics, pain and neurogenic dysfunction scientific podium short oral session 21. https://www.ics.org/2017/abstrct/451 (2017).

[8] Laurie B (2017). Infant Poty Training: A Gentle and Primeval Method Adapted to Modern Living.

[9] Blum NJ, Taubman B, Nemeth N (2004). Why is toilet training occurring at older ages? A study of factors associated with later training. J. Pediatr..

[10] Bakker E, Wyndaele JJ (2000). Changes in the toilet training of children during the last 60 years: The cause of an increase in lower urinary tract dysfunction?. BJU Int..

[11] Horn IB, Brenner R, Rao M, Cheng TL (2006). Beliefs about the appropriate age for initiating toilet training: Are there racial and socioeconomic differences?. J. Pediatr..

[12] Wu HY (2010). Achieving urinary continence in children. Nat. Rev. Urol..

[13] Choby BA, George S (2008). Toilet training. Am. Fam. Physician.

[14] Largo RH, Molinari L, von Siebenthal K, Wolfensberger U (1996). Does a profound change in toilet-training affect development of bowel and bladder control?. Dev. Med. Child Neurol..

[15] Mota DM, Barros AJ, Matijasevich A, Santos IS (2010). Longitudinal study of sphincter control in a cohort of Brazilian children. Jornal de Pediatria..

[16] Rugolotto S, Sun M, Boucke L, Calo DG, Tato L (2008). Toilet training started during the first year of life: A report on elimination signals, stool toileting refusal and completion age. Minerva Pediatr..

[17] Bakker E, Van Gool JD, Van Sprundel M, Van Der Auwera C, Wyndaele JJ (2002). Results of a questionnaire evaluating the effects of different methods of toilet training on achieving bladder control. BJU Int..

[18] Joinson C (2009). A prospective study of age at initiation of toilet training and subsequent daytime bladder control in school-age children. J. Dev. Behav. Pediatr..

[19] Yang SS, Zhao LL, Chang SJ (2011). Early initiation of toilet training for urine was associated with early urinary continence and does not appear to be associated with bladder dysfunction. Neurourol. Urodyn..

[20] Hellstrom AL (2000). Influence of potty training habits on dysfunctional bladder in children. Lancet.

[21] Stadtler AC, Gorski PA, Brazelton TB (1999). Toilet training methods, clinical interventions, and recommendations. American Academy of Pediatrics. Pediatrics.

[22] American Academy of Pediatrics. How to tell when your child is ready, 2nd edn. In *Guide to Toilet Training*, Vol. 1 (ed. Wolraich, M L.) 19–40. (Bantam Dell, Ebook ISBN 9780425285817, 2016)

[23] Van Herzeele C, Walle JV, Dhondt K, Juul KV (2017). Recent advances in managing and understanding enuresis. F1000Research..

[24] Yeung CK, Diao M, Sreedhar B (2008). Cortical arousal in children with severe enuresis. N. Engl. J. Med..

[25] Yeung CK (1995). Some new insights into bladder function in infancy. Br. J. Urol..

[26] Zhang YS (2016). Relationship between brain activity and voiding patterns in healthy preterm neonates. J. Pediatr. Urol..

[27] Neveus T (2010). Evaluation of and treatment for monosymptomatic enuresis: A standardization document from the International Children's Continence Society. J. Urol..

[28] 28Eaton A. Understanding habits and behaviours, 2nd ed. In *Stop Bedwetting in Seven Days,* Vol. 2, 30–35. (Troubador Publishing Ltd, 2012).

[29] Bender JM, She RC (2017). Elimination communication: Diaper-free in America. Pediatrics.

[30] Duong TH, Jansson UB, Holmdahl G, Sillen U, Hellstrom AL (2013). Urinary bladder control during the first 3 years of life in healthy children in Vietnam—A comparison study with Swedish children. J. Pediatr. Urol..

[31] Akis N, Irgil E, Aytekin N (2002). Enuresis and the effective factors—A case-control study. Scand. J. Urol. Nephrol..

[32] Rugolotto S, Sun M, Boucke L, Chen BB, Tato L (2008). Assisted infant toilet training: Is it time for a critical revision?. Med. Surg. Pediatr..

