# Supplementary material for: Disposable diaper overuse is associated with primary enuresis in children
Source: Sci Rep. 2020 Sep 1;10:14407. doi: 10.1038/s41598-020-70195-8 (PMC7462848; doi:10.1038/s41598-020-70195-8)
Supplement: Supplementary file 1 — Supplementary Information. [file 41598_2020_70195_MOESM1_ESM.pdf]

Disposable diaper overuse is associated with primary enuresis in children

Xing Li, Jianguo Wen, Tong Shen, Xiaoqing Yang, Songxu Peng, Xizheng Wang, Hui Xie, Xingdong Wu, Yukai Du

Supplementary 1 Study flowchart

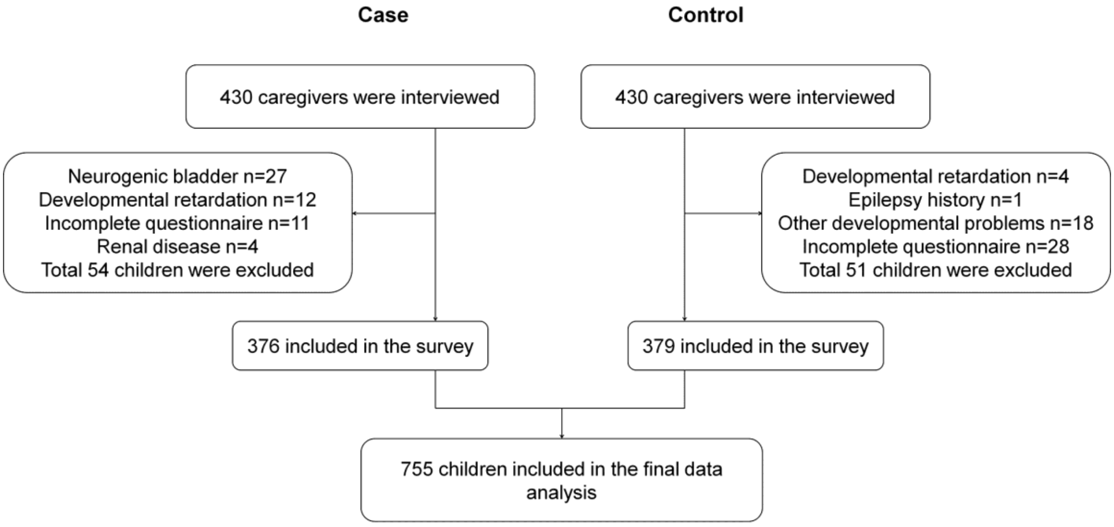

Supplementary 2

Children's Continence Questionnaire

No.□□□□□□

Dear parents, thank you for filling out this questionnaire. Please read the definitions below first.

●**Assistance Infant Toilet Training** (AITT): assistance infant toilet training is caregiver assisting and enabling the child to meet his or her basic cleanliness and health need for toileting from early infancy *via* verbal and non-verbal communication, to adopt a particular posture to help infant urine.

●**Toilet Training** (TT) (or continence training): put infants on the children's potty to practice urination or defecation. Repeat the process at least three times a day.

●**Nocturnal toilet training:** Caregivers introduced night training by purposeful removing diaper at night more than 3 times/week, go to toilet before go to bed, and wake the child up if find sign of restless or other signal for toilet at night if necessary.

●**Bed-wetting (enuresis)**: Your child does not consciously urinate after falling asleep and often wake up after urination, at least once a month.

General conditions of the child

1. Gender 1) Male □ 2) Female □;
2. Date of birth: □□□□YY□□MM□□DD
3. Your occupation ____;
4. Education□

   1) Junior high school and below 2) High school or vocational school

3) Junior college 4) Bachelor degree or above

5. Household registration□:

   1) urban household registration 2) rural household registration

6. The child was taken care of by□:

   1) parents 2) grandparents 3) maternal grandparents 4) A babysitter or others

Attitude and practices of toilet training

7. . Do you think it is helpful to assist infant toilet training within the age of 1?

   1) Yes □ 2) No □

   Please specify the reason for your selection: no more than 30 characters

8.. How old is your child when he/she was assisted to toilet training?□□

   1) Right after birth 2) After 3 days 3) After 1 month 4) After 3 months

   5) After 6 months 6) After the age of 1 7) After the age of 1.5

   8) After the age of 2 9) After the age of 2.5 10) After the age of 3

   ⑾ He/she was not assistant to toilet training.

9. Did your child use disposable diapers after birth? (multiple choice)

   1) Yes □ 2) No □

   3) He/she uses diapers in winter but does not use them in summer□

10. Did you use any of the following methods to help the child urinate/defecate while was using disposable diapers (multiple choices)

   1) Traditional practices□ (□times at daytime and □ times at nighttime)

   2) Cloth diapers are used in combination□(□ times at daytime and □ times at nighttime)

3) Other methods □(Urine car, split pants, urine isolation cushion and so on)

4) Only diapers are used

11. At what age did you start toilet training for continence during daytime?

1) Before 1 year □ 2) Before 18 months□ 3) Between 18-24 months

4) Between 25-30 months 5) After 30 months□ 6) Just waited□

12. At what age did you remove disposable diaper at daytime?

1) Before 1 year □ 2) Before 18 months□ 3) Between 18-24 months

4) Between 25-30 months 5) After 30 months□ 6) Just waited□

specifically, he/she did not use disposable diaper at daytime from the age of

□year(s) □□month(s)

13. Did the child signal to relieve himself? (Most of the time, the child indicates that he or she is urinating or defecating)

1) Yes□

He/she signals to defecate from the age of □year (s) □□month(s)

He/she signals to urinate from the age of □year (s) □□month(s)

2) No □

14. When did the child start urinating in a toilet seat or learn to squat down to urinate on his or her own? □year (s) □□month(s)

15. Did your child refuse to use a toilet to relieve himself/herself?

1) Yes ☐ 2) No ☐(skip to question 17)

If yes, when did the child refuse it?

1) Always 2) He/she refused to use toilets from the age of □year (s) □□month(s)

How long? □□month(s)

How did you handle this situation?

1) Insist that he/she uses the toilet or help him/her to urinate☐

2) Wait and see ☐ 3) Give up toilet training ☐

16. What attitude did you and your family hold towards the toilet training?

1) No training at all □

2) Toilet training occasionally□

3) A stern attitude towards training□

4) An encouraging attitude towards training□

5) Using interactive ways to help him/her poop and pee, receiving and responding to your child's signals or needs □

6) Cultivating regular habits of life, helping the child to poo and pee according to the regular daily schedule□

17. Did you train your child for urine continence during the night? Yes □, No ☐

If yes, what age did you introduce nighttime toilet training?

□year (s) □□month(s)

18. When did your child start napping without bed-wetting？

1) □year (s) □□month(s)

2) He/she is still wetting the bed.

19. When did your child stop wetting cloths or underwear during the day?

1) □year (s) □□month(s)

2) He/she is still wetting.

20. When did your child stop wetting during the night?

1) □year (s) □□month(s);

2) He/she is still wetting the bed. Frequency: □times a night,□ times a week or □ times a month;

Is it easy to wake up your child at night to urination? Yes □, No ☐

Does the child snore while sleeping? Yes □, No ☐

Do immediate family members (parents or siblings) have a history of bed-wetting? Yes □, No ☐

21. If the child has ever had any urination problems, please identify which one it is□:

1) Frequent urination 2) Urgent urination (cannot wait) 3) Leakage of urine

4) Hold pee by crossing legs, squatting or doing the “pee dance”

5) Urinary tract infection 6) Other problems

7) Not found

Frequency: Often □ Occasionally □

22. Does the child ever have any bowel movement problems, please identify:

1) Fecal incontinence (There were traces of excrement on his/her underwear)

2) Constipation (Defecate once every 2-3 days, and the stools are stiff)□

3) Anal fissure□ 4) Rectocele□ 5) Perianal redness and inflammation□

6) Hemorrhoids□ 7) Anal fistula□ 8) Stool Toilet Refusal □ 9)Stool hoding□

10) Defecating while standing□ ⑾Not found □

Birth conditions

23. Was the child born by vaginal delivery?

1) Yes □ 2) No ☐(dystocia□, Cesarean section□)

24. Week of gestation at birth: □□Weeks □Days; Weight at birth: □□Kg

25. Was the child breastfed after birth? □

1) Excluded breastfeeding 2) Formula feeding 3) Mixed feeding

26. How long have you been breastfeeding your child? □□month(s)

27. Was the child transferred to neonatal department after birth? 1) Yes □ 2) No ☐

If yes, what was/were the reason(s):1) Jaundice□ 2) Premature birth□ Newbone pneumonia□ 4) Infection□ 5) Hypoglycemia□ 6) Other (Please identify the reasons)

Thank you very much for your participation!

Your contact information:□□□□□□□□□□□
